# Cold Denaturation Unveiled: Molecular Mechanism of the Asymmetric Unfolding of Yeast Frataxin

**DOI:** 10.1002/cphc.201500765

**Published:** 2015-10-14

**Authors:** Domenico Sanfelice, Edoardo Morandi, Annalisa Pastore, Neri Niccolai, Piero Andrea Temussi

**Affiliations:** [a] Department of Basic and Clinical Neurosciences, Kings College LondonLondon, SE5 9RX, UK; [b] Department of Biotechnology, Chemistry and Pharmacy, University of Siena53100, Siena, Italy; [c] Department of Chemical Sciences, Università di Napoli Federico IIvia Cinthia, 80126, Napoli, Italy

**Keywords:** cold denaturation, electrostatic frustration, hydration, hydrophobic effect, protein folding

## Abstract

What is the mechanism that determines the denaturation of proteins at low temperatures, which is, by now, recognized as a fundamental property of all proteins? We present experimental evidence that clarifies the role of specific interactions that favor the entrance of water into the hydrophobic core, a mechanism originally proposed by Privalov but never proved experimentally. By using a combination of molecular dynamics simulation, molecular biology, and biophysics, we identified a cluster of negatively charged residues that represents a preferential gate for the entrance of water molecules into the core. Even single-residue mutations in this cluster, from acidic to neutral residues, affect cold denaturation much more than heat denaturation, suppressing cold denaturation at temperatures above zero degrees. The molecular mechanism of the cold denaturation of yeast frataxin is intrinsically different from that of heat denaturation.

It is well established that all proteins undergo cold-induced as well as heat-induced unfolding. The reason for this phenomenon was explained from fundamental thermodynamics principles by Privalov^1^ who postulated that cold denaturation is mainly driven by solvation of nonpolar side chains. Privalov suggested that cold denaturation is a phenomenon of all proteins, originating from the temperature dependence of the interaction of water with nonpolar side chains of protein residues. At variance with common belief, the free energy of hydration is negative and its absolute value increases as the temperature decreases. Consequently, at a critically low temperature, a compact folded protein is forced to unfold to expose the nonpolar groups of the hydrophobic core to water.^1^ Although quite convincing, this mechanism is nevertheless still unproven, debated, and often the object of heated controversy, with cold denaturation being attributed to a variety of physical causes, which, albeit not necessarily mutually exclusive, are mainly linked to the properties of water.2 Very little attention has been given to molecular aspects that might favor cold denaturation. Among the difficulties of collecting experimental proof for different mechanisms, the most relevant is probably that cold denaturation is seldom observed in wild-type proteins under physiological conditions without preventive destabilization.3 Here, we circumvented this difficulty by exploiting the properties of yeast frataxin (Yfh1), a protein that undergoes cold denaturation above the freezing point of water without artificial destabilization.^4^–^6^

In a recent study on the influence of salts on the stability of Yfh1, we suggested the presence of electrostatic frustration.^6^ Repulsion among spatially close negative charges located on adjacent elements of a secondary structure could lead to a weak point on the surface, facilitating the access of water molecules into the hydrophobic core. To unambiguously identify clusters of closely spaced acidic residues, we used extensive molecular dynamics (MD) simulations on Yfh1 (pdb id 2fql) and two frataxin orthologues of bacterial (CyaY, pdb id 1ew4) and human origin (hFrata, pdb id 1ekg), which do not show any tendency to undergo cold denaturation above the freezing point of water.^7^ MD provided a dynamic description of the protein surface that was much more informative than that obtained from the ‘static’ crystal structure. MD simulations of the three proteins were compared with the aid of an ad hoc-designed residue–residue interaction network,^8^,^9^ in which the nodes represent amino-acid residues. The thicknesses of the connecting lines show the persistence of short distances between the residues as a function of simulation time. The evolution of short-distance approaches among protein side chains was extracted from 50 ns trajectories by using a custom GROMACS tool to create a complete map of approaches (see the Supporting Information). To minimize the complexity of the final graphs, atoms used to extract the time evolution of distances were only included if the parent amino-acid residue was charged in at least one of the aligned frataxin orthologues. We filtered out all approaches occurring at a distance larger than 0.6 nm for more than 99 % of the entire MD trajectory (Figure S1). The analysis yielded an estimate of the time spent by each residue next to other residues of the protein at distances smaller than the given threshold. In Yfh1, there are two potential candidates for persistent electrostatic clusters on the protein surface. Both clusters are characterized by loose side chain–side chain approaches, that is, thin edges connecting the nodes (residues) inside the ovals (Figure 1 A**)**. The cluster within the green oval is unlikely to appreciably destabilize the tertiary structure, because all its residues belong to the same element of the secondary structure, that is, helix α1 (Figure 1 B). On the contrary, the residues of the yellow oval belong to two different strands of the beta sheet (β1 and β2).

**Figure 1:**
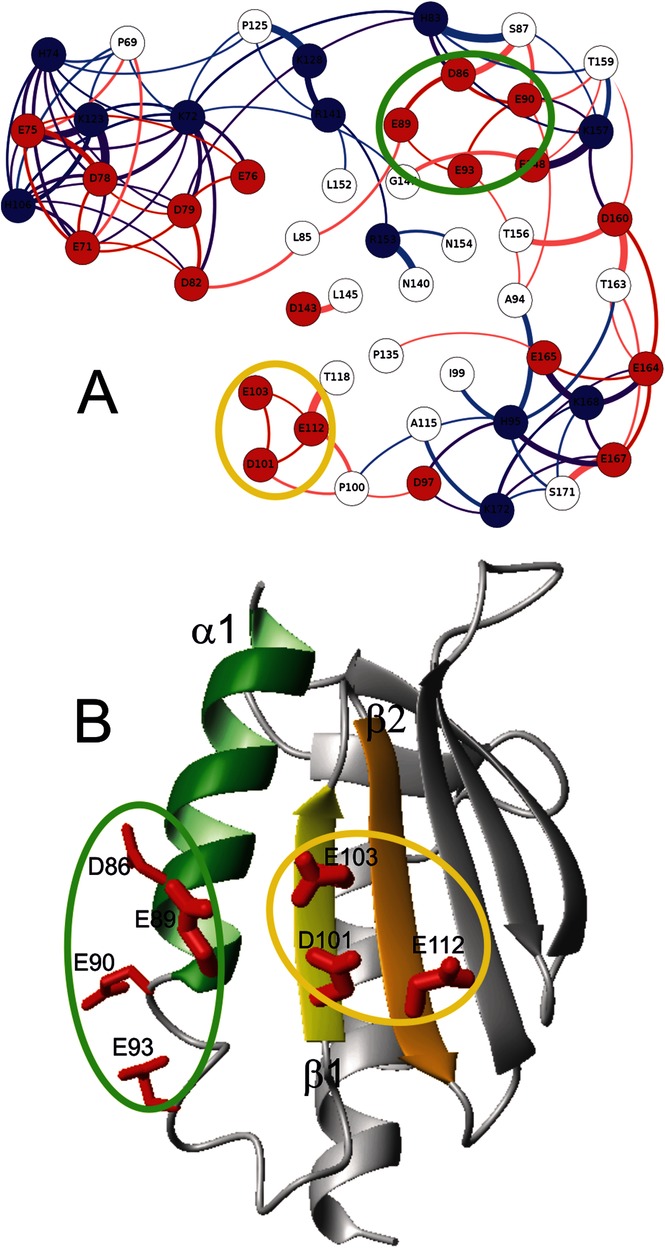
Clusters of negative charges on Yfh1. A) Graphic characterizing the evolution with time of relative spatial proximity of charged residues. Side chains are shown as nodes connected by edges, for which the thickness is proportional to the persistence of short-distance approaches. Circles corresponding to acidic residues are colored in red, those of basic residues are colored in blue. B) Ribbon representation of the structure of Yfh1 with the location of the two hypothetical clusters on the surface of Yfh1. The three elements of the secondary structure hosting clustered acidic residues are shown with different colors: green for helix α1 and two different shades of yellow for β1 and β2 strands. The molecular model was built with MOLMOL.^10^

To test the hypothesis of electrostatic frustration, we designed Yfh1 mutants that could suppress cold denaturation. In addition to D101, E103, and E112 (yellow oval), E89 (green oval) was also considered because it adds a new secondary structure element (helix α1) adjacent to the two β strands of the yellow oval residues. A comparison of the graphs of the three frataxins shows that the presence of T42 of CyaY (corresponding to E112 in Yfh1) and residues S126 and K135 in hFrata (corresponding to E103 and E112 in Yfh1) cause a marked closing up of the three residues of the β strands (Figure S2). We chose to mutate E103, E112, and E89 into serine residues because we wanted to relieve electrostatic frustration in Yfh1 without affecting the protein architecture or drastically altering the balance between hydrophilic and hydrophobic residues on the surface. We designed and expressed E89S Yfh1, E103S Yfh1, E112S Yfh1, the double mutants E89S/E103S Yfh1, E89S/E1112S Yfh1 and E103S/E112S Yfh1 plus the triple mutant E89S/E103S/E112S Yfh1.

Yfh1, at low ionic strength, exists as an equilibrium mixture of a folded and an unfolded species in slow exchange.^4^,^6^,11 In the ^15^N HSQC NMR spectrum of wild-type Yfh1 (Figure S3 A), the number of cross peaks is larger than those expected from the amino-acid sequence. The spectra of single mutants contain all of the peaks assigned to the folded species of Yfh1, but the outstanding feature is that peaks of the unfolded species (Figure S3) have decreased in intensity or are altogether absent, hinting at an overall stabilization of the proteins. The superposition of ^15^N HSQC NMR spectra of all mutants and the corresponding spectrum of wild-type Yfh1 shows that the increased stability is not accompanied by a significant change in protein architecture (Figure S4**)**. To compare the architecture of mutants at low salt concentration with the authentic folded form of Yfh1, only the spectrum of the wild-type Yfh1 in Figure S4 was recorded in the presence of 50 mm NaCl, which shifts the equilibrium toward a completely folded protein.11c The thermal stability of all constructs was gauged by using circular dichroism (CD) spectroscopy, monitoring the ellipticity at 222 nm as a function of temperature in the range 0–80 °C (Figure 2**)**. It is clear that each of the single mutations stabilizes Yfh1. The most striking feature of the thermograms (Figure 2 A**)** is that, although it is clear that each mutation stabilizes the protein, heat and cold denaturation are affected in a strikingly different way. The temperature of cold denaturation (*T*_c_) is shifted to lower values much more than the corresponding increases of heat denaturation (*T*_m_). Double and triple mutations are even more effective. Their thermograms do not show any sign of cold denaturation above the freezing point of water (Figure S5).

**Figure 2:**
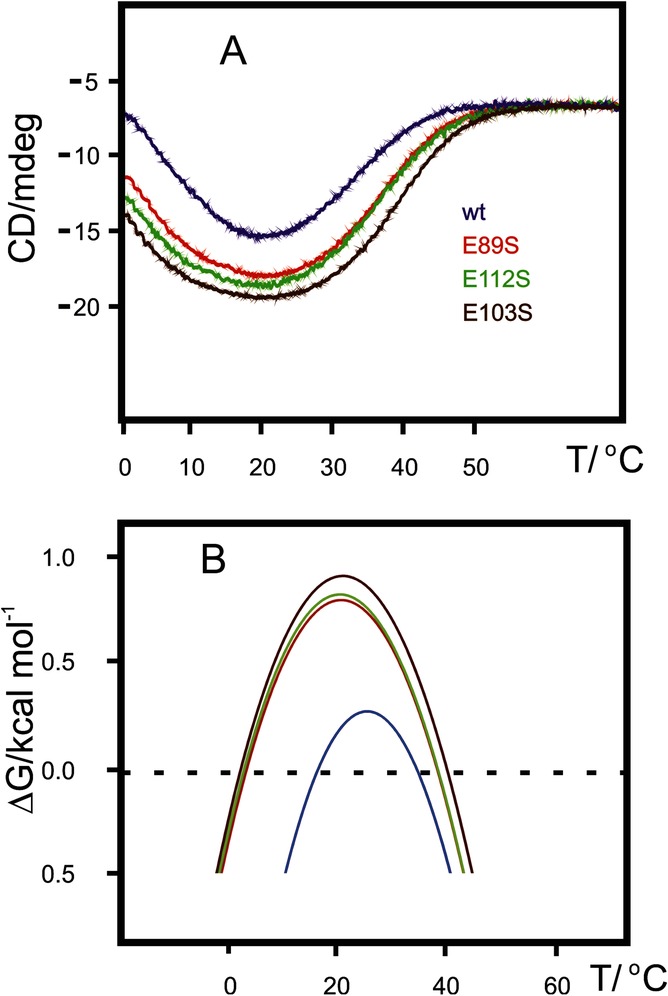
Comparison of the thermograms, obtained with CD spectroscopy, of wild-type Yfh1 with those of representative mutants. All solutions have a protein concentration of 10 μm in 10 mm HEPES buffer at pH 7.5. A) Variation in the intensity of the CD signal at 222 nm as a function of temperature for wild-type Yfh1, E89S Yfh1, E112S Yfh1, and E103S Yfh1. B) Stability curves corresponding to the thermograms of panel (A).

Relevant thermodynamic parameters (Table 1) were extracted from a plot of Δ*G* versus temperature, that is, from the protein stability curve (Figure 2 B).^12^ The values of *T*_m_ show only a moderate increase, as expected from mutations that only marginally affect a small surface area. However, the decrease in the cold denaturation temperature is strikingly large. Each of the three single mutations from an acidic residue (E) to a neutral one (S) leads to an increase in *T*_m_ of between 4 and 5 °C, but the corresponding values for *T*_c_ decrease by amounts of 13–14 °C, that is, approximately three times as much. The different influences of all mutations on heat and cold transitions hint at two intrinsically different mechanisms for high- and low-temperature unfolding. The simplest explanation for this behavior is that the low-temperature mechanism is linked to an intrinsically different interaction of water molecules with the hydrophobic core of the protein.^1^,^13^ Closing the gates to the entrance of water prevents the early onset of cold denaturation. This result is fully consistent with the different hydrations of the unfolded species at low or high temperatures that we recently reported.11

**Table 1:** Thermodynamic parameters^[a]^ of unfolding for Yfh1 and single mutants.

Construct	Δ*H* [kcal mol^−1^]	Δ*C*_p_ [kcal K^−1^ mol^−1^]	Δ*S* [kcal K^−1^ mol^−1^]	*T*_m_/*T*_c_ [°C]	Folding [%]
Wt	19.2	2.24	0.063	33.6/16.8	61
E89	29.5	1.73	0.095	37.2/4.1	80
E112	30.0	1.73	0.097	37.3/3.7	80
E103	31.3	1.69	0.10	38.9/3.1	83

[a] Δ*H*: change in enthalpy; Δ*C*_p_: change in heat capacity; Δ*S*: change in entropy.

We can, thus, conclude that stability frustration ensuing from the repulsion of negatively charged residues, while certainly not the only cause of decreased stability of yeast frataxin with respect to other orthologues,^7^ is a key step in promoting the cold denaturation of Yfh1. The interaction of water molecules with the side chains of hydrophobic residues at low temperature compensates the positive contributions of the enthalpic interactions, ultimately causing protein denaturation. These experimental data emphasize the importance of molecular mechanisms that are intrinsic to each protein in favoring cold denaturation and provide evidence that cold denaturation cannot only be attributed to water properties, as there is also an important contribution of the specific interactions within the protein. Although we cannot currently generalize our conclusions, owing to the paucity of examples of unbiased cold denaturation, we are presently working on verifying whether electrostatic frustration plays an important role in other cases.

## Experimental Section

### Sample Preparation

Natural-abundance and ^15^N-enriched Yfh1 and Yfh1 mutants were expressed in *E. coli* as described by He et al.^15^ Site-directed mutagenesis was performed by using desalted DNA primers from Integrated DNA Technologies (IDT).

### Far-UV CD Measurements

Far-UV CD spectra were run on a Jasco J-815 CD spectropolarimeter. Samples had a protein concentration of 10 μm in 10 mm HEPES buffer at pH 7.5. Thermal unfolding curves were obtained by monitoring the ellipticity at 222 nm with the Jasco CDF-4265/15 Peltier unit to control the temperature of the sample.

### NMR Spectroscopy

NMR spectra were acquired on a Bruker AVANCE spectrometer operating at 600 MHz ^1^H frequency. Typically, measurements were carried out in a 10 mm HEPES buffer, pH 7.5, using a 0.125 mm uniformly ^15^N-enriched protein. Water suppression was achieved by using WATERGATE,^16^ and HSQC experiments were used as described by Bax et al.^17^ Spectra were processed and zero-filled to the next power of two by using the NMRPipe program Delaglio et al.^18^

### Molecular Dynamics Simulations

Simulations were performed by using GROMACS and the AMBER force field, a dodecahedral box of equilibrated TIP3P water molecules, box boundaries of 0.7 nm, and chloride ions added to achieve global electric neutrality. The energy of the systems was minimized with 900 steps of conjugate gradients. During the initial 200 ps, an annealing procedure has been carried out to increase the box temperature from 0 to 300 K. Other simulation details and the generation of RIN graphs are described in the Supporting Information.

## Supporting Information

As a service to our authors and readers, this journal provides supporting information supplied by the authors. Such materials are peer reviewed and may be re-organized for online delivery, but are not copy-edited or typeset. Technical support issues arising from supporting information (other than missing files) should be addressed to the authors.
