# Supplementary material for: Cold Denaturation Unveiled: Molecular Mechanism of the Asymmetric Unfolding of Yeast Frataxin
Source: Chemphyschem. 2015 Oct 14;16(17):3599–602. doi: 10.1002/cphc.201500765 (PMC4676917; doi:10.1002/cphc.201500765)
Supplement: Supplementary file 1 [file cphc0016-3599-sd1.pdf]

CHEMPHYSCHEM

Supporting Information

Cold Denaturation Unveiled: Molecular Mechanism of the Asymmetric Unfolding of Yeast Frataxin

Domenico Sanfelice,[a] Edoardo Morandi,[b] Annalisa Pastore,*[a] Neri Niccolai,[b] and Piero Andrea Temussi*[a, c]

cphc_201500765_sm_miscellaneous_information.pdf

Supporting Information

MD and Graph generation.

The crystal structures of Yfh1 (pdb id 2fql), CyaY (pdb id 1ew4) and hFrata (pdb id 1ekg) were used, through Molecular Dynamics (MD) simulations, for building the RIN in 2D graphs. The X-ray structure of Yfh1 (2fql) corresponds to a Y73A mutant of Yfh1.[1] It was used because the structure of wild-type Yfh1 available in the PDB is not of sufficient quality, as recently demonstrated by Vilanova et al.[2] The single mutation is located on the very flexible N-terminal segment and does not affect directly any acidic residue.

GROMACS and the AMBER force field have been used to obtain 50 ns MD trajectories for the three solvated structures in a dodecahedral box of equilibrated TIP3P water molecules. The initial shortest distance between proteins and the box boundaries was set to 0.7 nm and chloride ions were added to achieve global electric neutrality. Afterwards, the energy of the systems was minimized with 900 steps of conjugate gradients. During the initial 200 ps an annealing procedure has been carried out to increase the box temperature from 0 to 300K. To achieve equilibration before the MD simulation, the three systems were subjected to short (20 ps) MD runs where protein atoms were restrained to their position and only solvent molecules were allowed to move. The protein-water system was simulated in the NPT ensemble at constant temperature (300 K) and pressure (1 atm). A weak coupling to external heat and pressure baths was applied, with relaxation times of 0.1 ps and 0.5 ps, respectively. Hydrogen covalent bonds were constrained using the LINCS algorithm and non-bonded interactions were computed using the PME method (27) with a grid spacing of 0.12 nm for electrostatic contribution and with a 0.9 nm cut-off for the van der Waals contribution. An integration time step of 2 fs was used and trajectory snapshots were saved every 1.0 ps. The time dependent calculation of distances between pairs of atoms is performed by *g_distmap*.

g_distmap: The GROMACS source tree has been modified in order to provide the compilation of the tool. The code is directly derived from *g_dist.c* and *gmx_dist.c* files belonging to the version 4.6.5 of the toolkit. *g_distmap* takes a trajectory file and two lists of atoms from an index file. It outputs a *xvg* file containing a table filled with interatomic

distances, where the columns are relative to the couples of atoms and the time dimension evolves with the rows. The legend contains the information about the couples of atoms.

The Euclidean distance evaluated for every combination of atoms and the results are stored in a standard XVG file. The list of atoms used as input to *g_distmap* is generated taking into account whether the amino acid residue is charged in at least one of the frataxin orthologs. Atoms selected to calculate relevant distances are the farthest side chain atoms least affected by internal rotation motions (Table S1).

Table S1. Atoms used as labels for side chain position along MD trajectory

aa	selected atom	aa	selected atom
A	CB	M	CE
C	SG	N	CG
D	CG[a]	P	CG
E	CD[a]	Q	CD
F	CZ	R	CZ[b]
G	CA	S	OG
H	CE	T	CB
I	CD	V	CB
K	NZ	W	NE1
L	CG	Y	OH

Atoms are named according to PDB file notations; in the case of R, E and D atoms have been selected to remove ambiguities due to a) O1/O2 and b) NH1/NH2 exchanges.

For every couple of atoms, the Euclidean distance is analyzed along the time dimension and a plot is computed. An example of such data processing, leading to peaks of different shape for different neighbors of Ser126 in hFrata, is shown in Figure S1.
The ratio of the area below the threshold distance and the total area of the peak is used to gauge the strength of the interaction in the network of charged residues. The network graph file was further generated by an *ad hoc* Python script and finally displayed using Gephi software.[3]

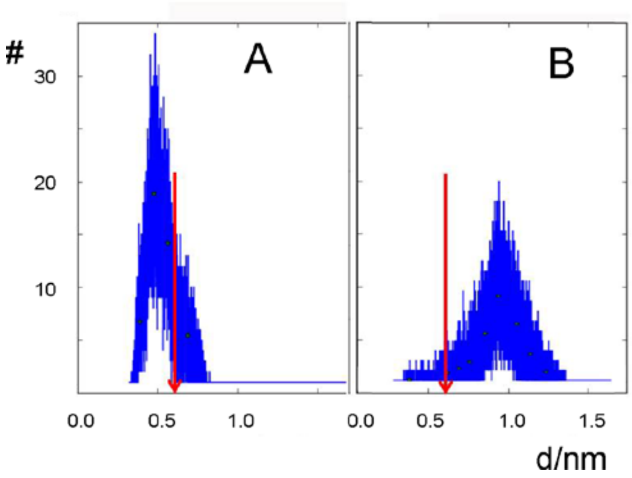

Figure S1: Evolution of the distance between representative side chain atoms of three hFrata residues (pdb id 1ekg). Each histogram height is proportional to the number (#) of distances binned during the calculation of each frame of the 50 ns MD trajectory (sampling rate was of 1 ps). A) Evolution of the distance between Ser126 OG and Asp124 CG. B) Evolution of the distance between Ser126 OG and Lys135 NZ. Red arrows are positioned at 0.6 nm, the threshold distance used in graph analysis.

Parsing the distance map file. The distance map file is parsed using a custom Python 3 script. The algorithm evaluates the binned distances for every couple and uses a filter method to remove irrelevant interatomic distances from the analysis. In particular, only the couples of atoms which spend at least 1% of the time of the trajectory within 0.6 nm of distance are kept, the others are filtered out. A network graph is generated using this information and generating a weighted interaction map between residues. For every residue a node is generated inside the graph, and the edge weight between a couple of nodes is proportional to the time a couple of representative atoms stay below the threshold of 0.6 nm. In detail, given a plot where the x axis represents the binned distances and the y axis is the number of frames the binned interatomic distance has a certain value, the weight of the edge for a certain couple of atoms is proportional to the ratio between the area of the plot below 6Å and the total area.

The final GEXF output file for the Gephi software is generated using the NetworkX Python package. To obtain visually comparable images, a specific procedure was developed: one of the GEXF files was selected as the template in Gephi modifying the positions of the nodes according to the Force Atlas layout. This generated a new temporary GEXF file. The node position was then modified in the other GEXFs according to the frataxin alignment, obtained by Clustal Omega.[4] When a graph contained a node not available in the template, this was manually positioned and the process repeated using the new template.

Visual alignment of network graphs. To obtain visually comparable results, another Python 3 script has been developed. Indeed, using the software Gephi, it is possible to place a graph in a 2D plane in order to get clear results. The Python script allows to copy the coordinates of the nodes to other graphs, keeping sequence-related residues at the same position. A multiple alignment between the sequences of the Frataxin orthologs is initially performed using Clustal Omega. The result is analyzed by the script, which, using a sample PDB from

the trajectory for every protein, performs a correlation between aligned atoms along different graph files. Using this information and the graph file generated by Gephi, the coordinates of the positioned nodes are copied to the nodes that still need to be correctly places. When a graph contains nodes that are not already placed because of an alignment gap between protein sequences, it is necessary to manually place these nodes and the script is run against the remaining graph files.

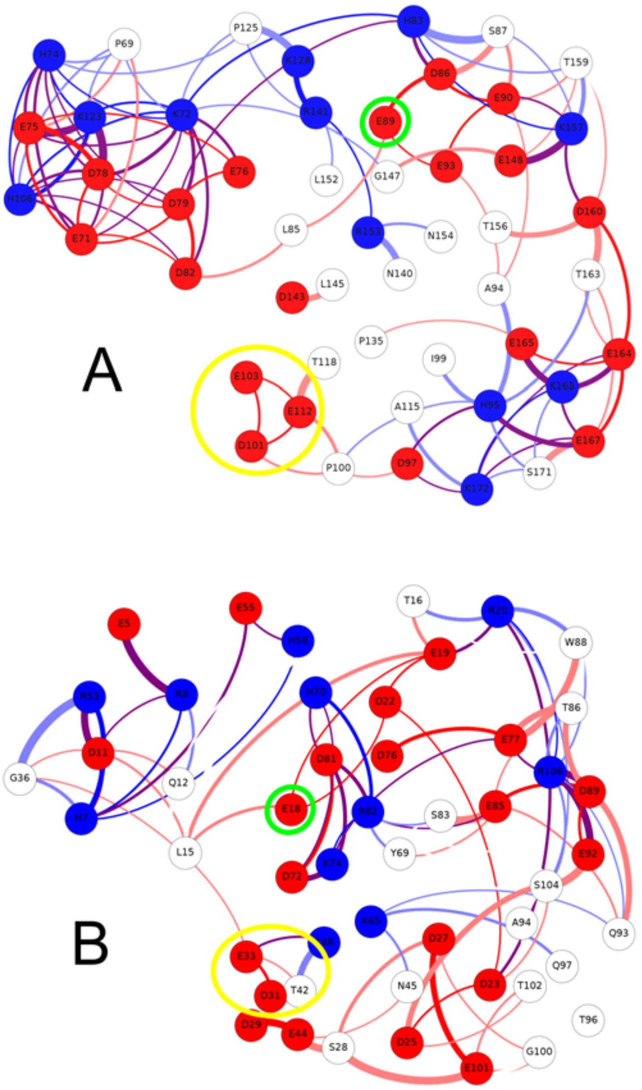

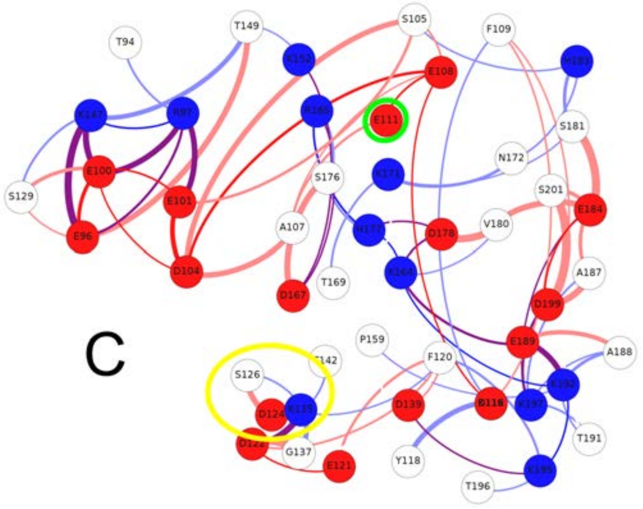

Figure S2. Comparison of the graphs of the frataxins from yeast (Yfh1, panel A), bacteria (CyaY, panel B) and humans (hFrata, panelC). Residues are represented with their one letter symbol inside a circle and the numbering corresponding to the chosen pdb id, *i.e* 2fql, 1ew4 and 1ekg for Yfh1, CyaY and hFrata respectively. Only residues that are charged in at least one of the frataxin orthologs are shown in the maps. Circles corresponding to acidic residues (D, E) are colored in red, those of basic residues (K, R, H) are colored in blue. All remaining residues are inside blank circles. The color of edges reflects those of connected nodes. The yellow oval of Yfh1 comprises D101, E103 and E112, that of CyaY contains two negative residues (D31 and E33) and T42; which are flanked by a positive residue (K48). The residues of hFrata corresponding to the yellow oval of Yfh1 comprise one negative residue (D124), one neutral residue (S126) and one positive residue (K135).

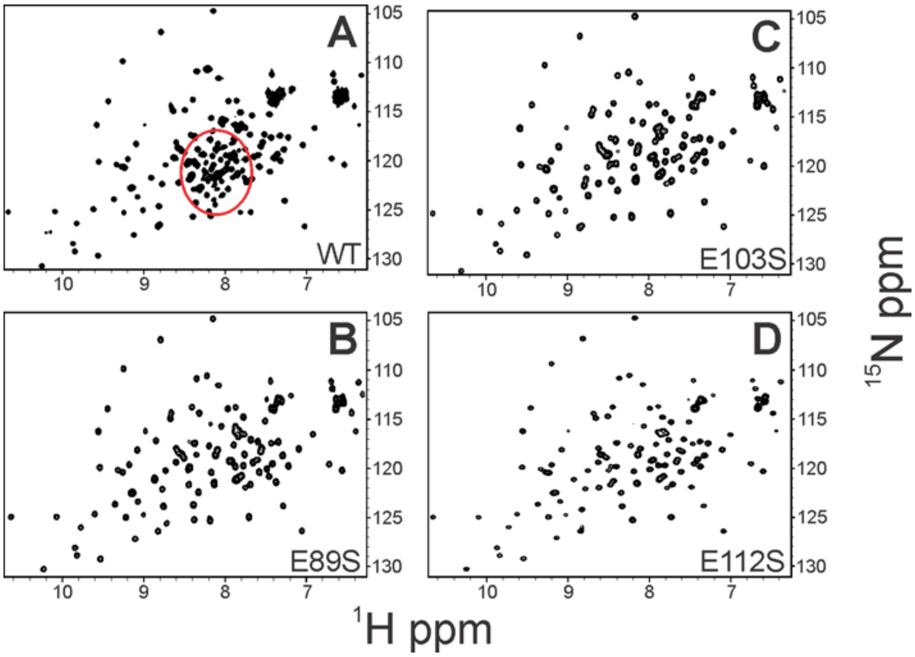

Figure S3. Comparison of the ^{15}N HSQC NMR spectra of wild type Yfh1 and of three single mutation constructs. All solutions are 125 μM in 10 mM HEPES buffer at pH 7.5. A) ^{15}N HSQC NMR spectrum of wild type Yfh1. B, C, D) ^{15}N HSQC NMR spectra of E103S, E89S and E112S Yfh1 respectively. All spectra were recorded at 25 °C.

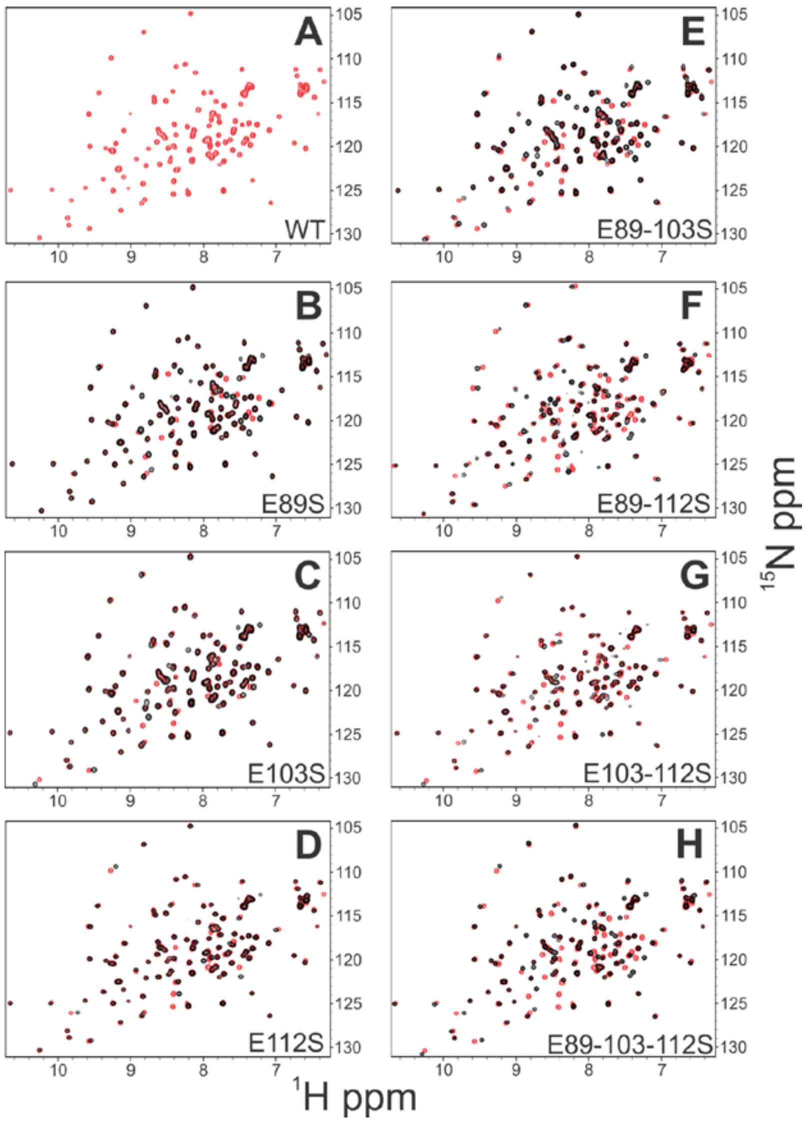

Figure S4. Comparison of the ^{15}N HSQC NMR spectra of wt Yfh1 and of all mutants. A) spectrum of wild type Yfh1 in 10 mM HEPES buffer with the addition of NaCl to a final concentration of 50 mM. B-H) spectra of all mutants studied; all solutions of the mutants constructs are 125 μM in 10 mM HEPES buffer at pH 7.5 without any addition of salt.
To make the comparison easier, the spectrum of wild type Yfh1 shown in panel A was recorded in a solution containing 50 mM NaCl that shifts the equilibrium toward a completely folded protein. The spectra of mutants at low salt are very similar to that of the wt in medium ionic strength. The cross peaks of the wild-type protein[11c] are colored in red, those of the mutants are in black. All spectra were recorded at 25 °C.

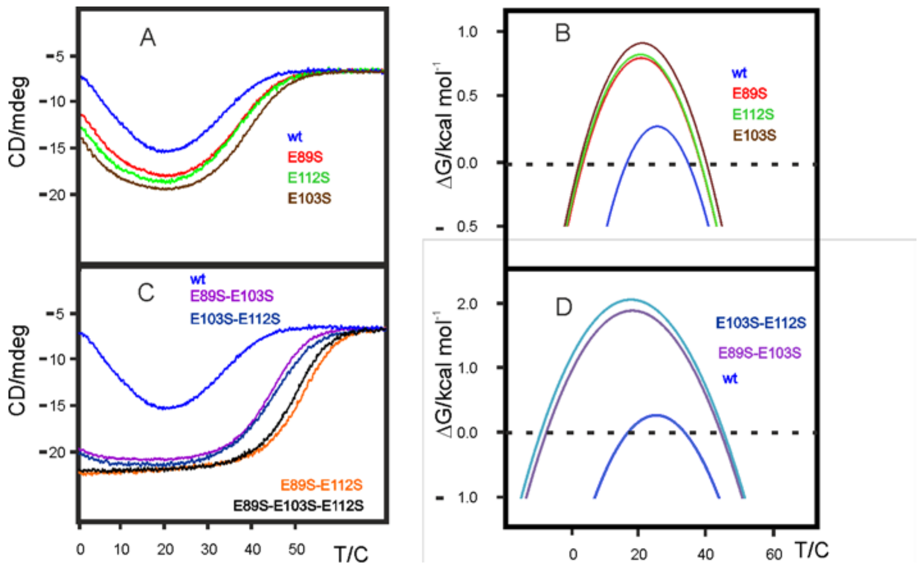

Figure S5. Comparison of the thermograms, obtained with CD spectroscopy, of wild type Yfh1 with those of all mutants. All solutions have a protein concentration of 10μM in 10 mM HEPES buffer at pH 7.5. A) Variation of the intensity of the CD signal at 222 nm as a function of temperature for wild type Yfh1 and E89S Yfh1, E112S Yfh1 and E103S Yfh1. B) Stability curves corresponding to the thermograms of panel A. C) Variation of the intensity of the CD signal at 222 nm as a function of temperature for wild type Yfh1 and E89S-E103S Yfh1, E103S-E112S Yfh1, E89-E112S Yfh1 and E89S-E103S-E112S. D) Stability curves corresponding to the thermograms of panel C.

The thermograms of E89/E103S Yfh1 and E103S/E112S Yfh1 hint at higher populations of the folded species at room temperature, but retain also some curvature typical of incipient low temperature unfolding as the temperature of 0°C is approached (Figure S5 C). On the contrary, the thermograms of E103S/E112S Yfh1 and of E89/E103S/E112S Yfh1 (Figure S5 C), characterized by a flat low temperature stretch, are indistinguishable from those of the more stable frataxin orthologs.

----

[1] T. Karlberg, U. Schagerlöf, O. Gakh, S. Park, U. Ryde, M. Lindahl, K. Leath, K., E. Garman, G. Isaya, S. Al-Karadaghi, *Structure* **2006**, *14*, 1535.

[2] B. Vilanova, D. Sanfelice, G. Martorell, P. A. Temussi, A. Pastore, *Front Mol Biosci.,* **2014**, *1*, 13.

[3] M. Bastian, S. Heymann, M. Jacomy M. *International AAAI Conference on Weblogs and Social Media* (2009).

[4] F. Sievers, A. Wilm, D. G. Dineen, T. J. Gibson, K. Karplus, W. Li, R. Lopez, H. McWilliam, M. Remmert, J. Söding, J. D. Thompson, D. G. Higgins, *Molecular Systems Biology* **2011**, *7*, 539.
